# Worldwide use of the first set of physical activity Country Cards: The Global Observatory for Physical Activity - GoPA!

**DOI:** 10.1186/s12966-018-0663-7

**Published:** 2018-03-27

**Authors:** Andrea Ramirez Varela, Deborah Salvo, Michael Pratt, Karen Milton, Katja Siefken, Adrian Bauman, Harold W. Kohl, I-Min Lee, Gregory Heath, Charlie Foster, Kenneth Powell, Pedro C. Hallal

**Affiliations:** 1 Post-Graduate Program in Epidemiology, Federal University of Pelotas, Pelotas, Brazil; 2 The University of Texas Health Science Center at Houston (UTHealth), School of Public Health in Austin, Austin, USA; 3 Center for Nutrition and Health Research, National Institute of Public Health of Mexico, Cuernavaca, Mexico; 4 San Diego School of Medicine, University of California, San Diego, USA; 5 Norwich Medical School, University of East Anglia, Norwich, UK; 6 School of Health Sciences, University of South Australia, Adelaide, Australia; 7 Sydney School of Public Health, University of Sydney, Sydney, Australia; 8 The University of Texas at Austin, Austin, USA; 9 Harvard Medical School, Harvard T.H. Chan School of Public Health, Brigham and Women’s Hospital, Boston, USA; 10 College of Medicine Chattanooga, University of Tennessee, Chattanooga, USA; 11 University of Bristol, Bristol, UK; 12 Atlanta, USA

**Keywords:** Global health, Process evaluation, Public health, Surveillance data methods

## Abstract

**Background:**

The work of The Global Observatory for Physical Activity-GoPA! is the first global effort to compile standardized country-level surveillance, policy and research data for physical activity in order to better understand how countries and regions address promoting physical activity. GoPA! developed standardized country-specific physical activity profiles (“Country Cards”) to summarize country-level data through 2013. The aim of this study was to assess use of the Country Cards, identify the factors associated with their use, and develop recommendations for supporting country-level physical activity promotion.

**Methods:**

Cross sectional internet-based survey conducted between August–October 2016. Target study participants were national physical activity leaders and advocates in academia, government and practice from the GoPA! countries, and members of the International Society of Physical Activity and Health. A Country Card use composite score was created based on the diversity and frequency of use. Statistical analyses on the associations between the composite score and respondent characteristics, country characteristics, barriers and opinions were conducted (including descriptive analyses and a logistic regression with robust standard errors).

**Results:**

One hundred forty three participants from 68 countries completed the survey. Use of the Country Cards was associated with being part of the GoPA! network, knowing about the Country Cards, and on the stage of country capacity for physical activity promotion. Country Card knowledge varied by country income group, region and the country specific context. More diverse and frequent use of the cards (highest tertile of the composite score for use) was associated with: 1. Being a country contact vs general participant (OR 18.32–95% CI 5.63–59.55, *p* = 0.002), and 2. Collaborating with a government representative working in NCDs on a monthly or more frequent contact vs less frequent contact (OR 3.39–95% CI 1.00–11.54, *P* < 0.05).

**Conclusions:**

For the Country Cards to have a broader impact, GoPA! will need to widen its reach beyond the academic sector. With further refinement of the cards, and training in their implementation, they could be an important tool for advancing country capacity for contextually-relevant strategies, actions and timelines for PA promotion.

**Electronic supplementary material:**

The online version of this article (10.1186/s12966-018-0663-7) contains supplementary material, which is available to authorized users.

## Background

In 2012, in response to the global pandemic of physical inactivity [1, 2] the Global Observatory for Physical Activity - GoPA! http://www.globalphysicalactivityobservatory.com/ [3] was created. At the time, information on the global picture of how well countries across the world were progressing on promoting physical activity was quite limited. Specifically, little standardized information was available on surveillance, policy and research on physical activity [4]. The work of GoPA! is the first attempt to compile standardized country-level data on surveillance, policy and research to better understand how countries and regions are faring in promoting physical activity [5–7]. GoPA! also aims to enhance evidence-informed decision making and to produce meaningful public health actions and policies worldwide to curb the inactivity pandemic. The first step towards fulfilling this goal was the development of standardized country-specific physical activity profiles (“Country Cards”) to summarize country-level data up to 2013, and to provide comparable indicators for: demographics, physical activity prevalence, existence of physical activity surveillance systems, policy and research indicators.

Between 2014 and 2016, GoPA! gathered information for 217 countries. Among these, 139 (64%) countries had full, valid and approved (by a country contact) data for all indicators, covering 84% of the 2013 world population. The methods for creating this first standardized set of country cards, and the results by country for surveillance, research and policy indicators have been previously published [7]. These data are also summarized in the “1st Physical Activity Almanac” [3].

An important finding noted in these publications is a significant positive correlation between research productivity, regular surveillance, and standalone physical activity policy indicators [7], suggesting that progress in any of these three areas may stimulate progress in the other two [7]. Previous evidence supporting the importance of physical activity surveillance and policy indicators highlights the need for monitoring levels of physical activity in a country as a key first step in “making the case” for developing a national physical activity strategy and plan [5]. Translational research demonstrates the importance of research evidence for guiding optimal policy choices for population health [8–11].

**Fig. 1:**
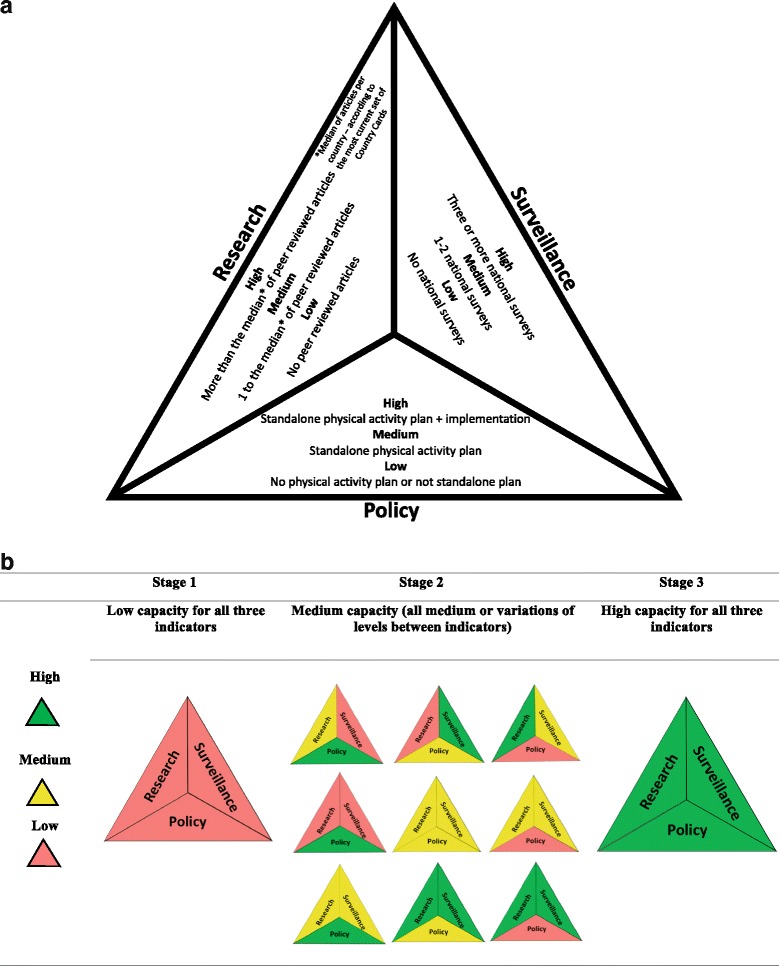
Pyramid and stages of country-level capacity for physical activity promotion based on the Country Card indicators. 1. **a** GoPA! pyramid for country-level capacity for physical activity promotion 1. **b** Stages of country-level capacity for physical activity promotion based on GoPA! Country Card indicators

The aforementioned evidence guided the development of a GoPA! conceptual model for country-level capacity for physical activity promotion, including periodic surveillance, implementation of physical activity policy, and research productivity as the three pillars (Fig. 1).

The aims of this study were to assess the use of the first set of GoPA! Country Cards and to identify the factors associated with their use. The results of this study will guide the development of future sets of Country Cards and future assessment of country-level progress towards reducing physical inactivity, as well as informing country-level physical activity promotion based on the first set of Country Cards.

## Methods

### Study design

This was a cross sectional internet-based survey conducted between August–October 2016. The target study participants were national physical activity leaders and advocates in academia, government and practice from the 139 GoPA! countries with complete Country Cards available by August 2016 (list of GoPA! country members in Additional file 1), members of the International Society of Physical Activity and Health, and subscribers to the GlobalPAnet E-Bulletin.

### Sampling, recruitment and data collection

ISPAH is a professional member-based society which aims to “promote physical activity as a global health priority through excellence in research, education, capacity building and advocacy” [12]. ISPAH supports a website and fortnightly E Bulletin called GlobalPAnet to share knowledge on physical activity related research, practice and policy. Both members and non-members of ISPAH can subscribe to GlobalPAnet. All GoPA! Country Contacts, ISPAH members, and GlobalPAnet subscribers were invited to participate in the study. GoPA! is an ISPAH Council, thus there was overlap between the GoPA!, ISPAH, and GlobalPAnet mailing lists. As GlobalPAnet was the largest mailing list, this was used to estimate the response rate of participants who were non-GoPA! Country Card contacts.

A questionnaire was sent via an electronic data collection system (online questionnaire using the Survey Monkey platform) and was emailed to the representatives of each of the 139 GoPA! countries with Country Cards available up to November 2016. A more general email was sent to the wider e-mail list (more than 1700 email addresses). During a two-month period, four reminders were sent using email and social media (Twitter and Facebook). No confidential, private, or sensitive information was collected and the survey was anonymous, therefore no signed informed consent was required. This study was approved by the Research Ethics Committee of the Faculty of Physical Education (n° 522.064) at the Federal University of Pelotas, Brazil. CAAE n° 67102116.0.0000.5313.

### Measures

The online survey was designed in May 2016 and included eight questions related to Country Card performance/implementation divided in three blocks: 1) Country Card use and frequency of use; 2) Country Card users and country characteristics; and, 3) Perceived barriers and opinions about the Country Cards. The survey was revised and approved by the GoPA! steering committee (http://www.globalphysicalactivityobservatory.com/) and the country contacts from the UK (experts in evaluation of physical activity public health programs) from June to July 2016.

### Country card use and frequency of use

Questions on the survey related to country card use included the following: 1-Presentation to different audiences (colleagues; students; academic societies; local, state or federal government; non-government representatives; or mass media representatives); 2-How the information was conveyed to these audiences (congresses; scientific events; in scientific publications; fund raising proposals; and policy briefs); and, 3-The motivation/rationale behind the communication with different audiences (to advocate for a national surveillance system or national physical activity plan). The frequency of communication with these audiences was defined as more frequent (at least once a month) or less frequent (less than once a month). A **Country Card composite use score** was created as the main dependent variable, with a maximum score of 48 that combined two aspects: 1- diversification of use (12 possible uses for the Country Card – 1 point for each use); and, 2- frequency of use (“never”-0 points, “less than monthly”-1 point, “approximately monthly”-2 points, “approximately weekly”-3 points and “daily or almost daily”-4 points). The score was divided into tertiles and a dichotomous variable was created (highest tertile of use vs lowest tertiles as the reference category).

### Country card users

The characteristics of the Country Card users included: 1-main area of work (academia, non-academia, local, state or federal government, non-government, other); 2-being a GoPA! Country representative; and, 3- interaction and frequency of contact (meetings, email or phone calls) with other sectors (physical activity researchers, government representatives, non-government representatives, and GoPA! country contacts).

### Country level characteristics

Country characteristics included: 1- Region classification according to the World Health Organization (EURO - European Regional Office of the World Health Organization; AFRO - African Regional Office of the World Health Organization; PAHO - Pan American Health Organization of the World Health Organization; EMRO - Eastern Mediterranean Regional Office of the World Health Organization; WPRO - Western Pacific Regional Office of the World Health Organization; SEARO - South-East Asia Regional Office of the World Health Organization); 2- Income level classification according to the World Bank; Country Card national indicators of deaths related to physical inactivity, surveillance, policy, research and physical activity prevalence estimates.

### Perceived barriers and opinions about the country cards

The extent of agreement or disagreement with potential barriers to Country Card use and feedback on the Country Cards was assessed. Barriers included: 1-presentation of already known information; 2-unclear purpose of the card; and 3-unclear on the recommended strategy to identify and reach relevant partners, decision makers and/or stakeholders. Feedback included: 1-the card was helpful for making the case for physical activity promotion and the feedback participants received about the Country Cards (open ended question). Finally, the respondent ranked the importance/relevance on a scale from 1 to 5 (5 being most important) of the indicators presented in the Country Cards (deaths related to physical inactivity, surveillance, policy, research and physical activity prevalence estimates) to describe the status of physical activity at the national level.

### Analyses

Country was the unit of analysis and the main outcome variable was the *Country Card use composite score* in the highest tertile vs lowest tertiles as reference categories. Statistical analyses were performed in STATA version 12.0. Descriptive analyses were conducted for the sample and the absolute and relative frequencies of dependent and independent variables were calculated.

Bivariate analyses were conducted on the associations between the Country Card use composite score in the highest tertile and respondent and country characteristics and barriers and opinions, using the heterogeneity chi-square test. Possible confounding variables were identified as those associated (*p* < 0.20) with both the exposure and at least one outcome variable, and were included in the final multivariate analytical models. A logistic regression with robust standard errors was used to obtain adjusted effect estimates (including confounding factors). The *p*-value for statistical significance was set at < 0.05 in the final model. Open-ended questions were reviewed by an expert in qualitative analyses.

## Results

During the two-month data collection period, 143 participants from 68 countries completed the survey (Table 1). Respondents included GoPA! country contacts (37.1%) and Global PA Network/ISPAH participants (who were not GoPA! country contacts) (62.9%). The GoPA! country contacts response rate was 38.1% (53/139). Additional file 1, shows GoPA! had 139 country members by November 2016. GlobalPANetwork /ISPAH member’s response rate was 5.3% (90/1703).

**Table 1:** Respondent characteristics

	Total		GoPA! Country Contacts		ISPAH respondents (not GoPA! Country Contacts)	
	n^a^	%	n^a^	%	n^a^	%
Participation in the survey	143	100.0	53	37.1	90	62.9
Main area of work						
Academia (universities, schools, societies or institutions)	117	81.8	44	83.0	73	81.1
Government	13	9.1	3	5.7	10	11.1
Other	13	9.1	6	11.3	7	7.8
Frequency of contact with:						
Researchers by any of the three means (emails, meetings, phone calls)						
Contact using any mean at least once a month	119	83.2	44	83.0	75	83.3
Contact using the three means less than once a month	24	16.8	9	17.0	15	16.7
Contact with government representatives working in physical activity promotion at any of the levels						
Contact with any representative at least once a month	66	46.2	32	60.4	34	37.8
Contact with the representatives less than once a month	77	53.9	21	39.6	56	62.2
Contact with government representatives working in non-communicable diseases NCD’s						
Contact with any representative at least once a month	56	60.8	28	52.8	28	31.1
Contact with the representatives less than once a month	87	39.2	25	47.2	62	68.9
Non-government organization representatives working in physical activity promotion						
More frequent (at least once a month)	66	46.2	32	60.4	34	37.8
Less frequent (less than once a month)	77	53.9	21	39.6	56	62.2
International organizations representatives working in physical activity promotion						
More frequent (at least once a month)	56	39.2	28	52.8	28	31.1
Less frequent (less than once a month)	87	60.8	25	47.2	62	68.9
GoPA! Country Contacts						
More frequent (at least once a month)	34	23.9	21	39.6	13	14.6
Less frequent (less than once a month)	108	76.1	32	60.4	76	85.4
World WHO region^b^						
AFRO	12	8.4	6	11. 3	6	6.7
EMRO	5	3.5	26	49.1	26	28.9
EURO	52	36.4	3	5.7	2	2.2
PAHO	43	30.1	11	20.8	32	35.6
SEARO	5	3.5	3	5.7	2	2.2
WPRO	26	18.2	4	7.6	22	24.4
Country-level Income group						
High Income	97	67.8	32	60.4	65	72.2
Upper Middle Income	30	21.0	11	20.8	19	21.1
Lower Middle Income	10	7.0	7	13.2	3	3.3
Low Income	6	4.2	3	5.7	3	3.3

^a^n does not add to total value of 143 due to missing data

^b^EURO - European Regional Office of the World Health Organization; AFRO - African Regional Office of the World Health Organization; PAHO - Pan American Health Organization of the World Health Organization; EMRO - Eastern Mediterranean Regional Office of the World Health Organization; WPRO - Western Pacific Regional Office of the World Health Organization; SEARO - South-East Asia Regional Office of the World Health Organization

### Participant characteristics

Survey respondents were mostly from EURO (36.4%) and PAHO (30.1%) followed by Western Pacific (WPRO) (18.2%), Africa (AFRO) (8.4%), Eastern Mediterranean (EMRO) (3.5%) and, South East Asia (SEARO) (3.5%). The majority of participants were from high- and upper middle-income countries (89%) (Table 1 Most of the participants reported that they worked in academia (81.8%); and most frequently had contact (monthly or more often) with physical activity researchers by email (84.2%) (Table 2).

**Table 2:** Country Cards uses referred by respondents

	Total		GoPA! Country Contacts		ISPAH respondents (that are not GoPA! Country Contacts)	
	n^a^	%	n^a^	%	n^a^	%
Use of the card (any of the 12 possible uses)						
One or more uses (mean & SD)^b^	143	6.4 (4.3)	53	8.9 (4.4)	90	4.8 (3.4)
Never (mean & SD)^b^		7.5 (4.3)		4.6 (4.1)		9.2 (3.4)
Showed/ described/explained the Country Cards to colleagues (Use 1)						
More frequent (at least once a month)	39	27.3	25	47.2	14	15.6
Less frequent (less than once a month)	104	72.7	28	52.8	76	84.4
Showed/ described/explained the Country Cards to academic societies representatives working in non-communicable diseases NCD prevention and physical activity promotion (Use 2)						
More frequent (at least once a month)	27	19.0	18	34.0	9	10.1
Less frequent (less than once a month)	115	81.0	35	66.0	80	89.9
Showed/ described/explained the Country Cards to non-government organizations representatives (Use 3)						
More frequent (at least once a month)	16	11.2	12	22.6	4	4.4
Less frequent (less than once a month)	127	88.8	41	77.4	86	95.6
Showed/ described/explained the Country Cards to mass media representatives (Use 4)						
More frequent (at least once a month)	11	7.7	9	17.0	2	2.2
Less frequent (less than once a month)	132	92.3	44	83.0	88	97.8
Showed/ described/explained the Country Cards to students (Use 5)						
More frequent (at least once a month)	30	21.0	21	39.6	9	10.0
Less frequent (less than once a month)	113	79.0	32	60.4	81	90.0
Showed/ described/explained the Country Cards in congresses or scientific events (Use 6)						
More frequent (at least once a month)	9	6. 3	7	13. 2	2	2. 2
Less frequent (less than once a month)	134	93. 7	46	86. 8	88	97.8
Included/described/explained the Country Cards in a scientific publication (Use 7)						
More frequent (at least once a month)	14	9.9	11	21.6	3	3.3
Less frequent (less than once a month)	127	90.1	40	78.4	87	96.7
Included/described/explained the Country Cards as part of a fund raising proposal (Use 8)						
More frequent (at least once a month)	4	2.8	4	7.7	0	0.0
Less frequent (less than once a month)	137	17.7	48	28.9	89	100.0
Included/described/explained the Country Cards in a policy brief (Use 9)						
More frequent (at least once a month)	7	4.9	7	13.5	0	0.0
Less frequent (less than once a month)	135	95.1	45	86.5	90	100.0
Presented/described/used the data presented in the Country Cards to advocate for a national surveillance system (Use 10)						
More frequent (at least once a month)	8	5.6	8	15.0	0	0.0
Less frequent (less than once a month)	135	94.4	45	84.9	90	100.0
Presented/described/used the data presented in the Country Cards to advocate for a national physical activity plan (Use 11)						
More frequent (at least once a month)	15	10. 5	14	26.4	1	1.1
Less frequent (less than once a month)	128	89.5	39	73.6	89	98.9
Showed/ described/explained the Country Cards to government representatives at any level (local, state, federal) (Use 12)						
More frequent (at least once a month)	12	8.5	10	19.2	2	2.2
Less frequent (less than once a month)	130	91.6	42	80.8	88	97.8

^a^n does not add to total value of 143 due to missing data

^b^Mean and standard deviation

### Country card diversification of use

There was a broad range of knowledge and use of the Country Cards among the survey respondents. The mean number of ways in which the card was used was 6.4 (SD 4.3) out of the 12 possibilities. Country contacts had a mean number of uses of 8.9 (SD 4.4) and non-Country card contacts a mean of 4.8 uses (SD 3.4). When analyzing mean use by region, PAHO was the region with the highest mean total use (total 7.1 (SD 4.3), followed by EMRO (total 7.0, SD 5.8), EURO (total 6.6, SD 4.4), SEARO (total 5.6, SD 5.6), WPRO (total 5.3, SD 3.9), and AFRO (total 5.3, SD 3.4). In all regions, Country Card use was greater among country contacts than non-Country Card contacts. The ways in which the Country Cards were disseminated ranged from making a reference to the Country Cards within doctoral theses to discussing the results with the Ministry of Health.

### Country card frequency of use

The cards were most frequently shown (on a monthly or more often basis) to colleagues (27.3%), students (21.0%), academics in non-communicable disease (NCD) prevention and/or physical activity promotion (19.0%), and government representatives at the state level (17.7%). Country Cards were most often (at least once) presented in scientific events (37.8%) or included in scientific publications (30.5%), policy briefs (28.8%) or fund raising proposals (20.6%). Approximately one third of the participants (31.5%) used the Country Card to advocate for physical activity surveillance and policy at the national level (Table 2).

### Factors associated with country card composite use score

The following characteristics were significantly (*p* < 0.05) associated with *Country Card composite use score* in the bivariate analysis: 1- positive associations with country contact status; having contact with researchers, government representatives at local, state and federal levels and representatives from international organizations; and, Country Cards indicators of policy, surveillance and research. Negative associations were found with the barriers to Country Cards use.

In the adjusted model, the use of the Country Card in the highest tertile of the composite score was positively and significantly associated with: being a country contact vs non-country contact (OR 18.32–95% CI 5.63–59.55, *p* = 0.002); A monthly or more frequent contact with a government representative working in NCDs vs less frequent contact (OR 3.39–95% CI 1.00–11.54, *P* < 0.05). Agreeing that the card was useful for making the case for physical activity was positively and significantly associated with composite use scores in the highest tertile when compared to the users who thought it was not useful (OR 32. 5–95% CI 5.22–202.21, *P* < 0.001). Table 3 presents the factors associated with the Country Card composite use score according to respondent characteristics.

**Table 3:** Factors associated with the Country Card composite score use in the highest tertile according to respondent’s characteristics

Adjusted model (Highest tertile of use vs lowest tertiles of use)^a^						
	n	%	OR (95% CI)^a^			*p*-value
Main area of work						
Academia (universities, schools, societies or institutions)	40	85.1	1.00			0.283
Government	2	4.3	0.34	0.04	3.22	
Other	5	10.6	4.82	0.87	26.71	
Country Contact						
Yes	36	76.6	18.32	5.63	59.55	0.002
No	11	23.4	1.00			
Contact with researchers by any of the three means (emails, meetings, phone calls)						
Contact using any mean at least once a month	33	70.2	1.29	0.41	4.08	0.658
Contact using the three means less than once a month	14	29.8	1.00			
Contact with government representatives working in physical activity promotion at any of the levels						
Contact with any representative at least once a month	43	91.5	1.47	0.33	6.66	0.612
Contact with the representatives less than once a month	4	8.5	1.00			
Contact with government representatives working in NCD’s						
Contact with any representative at least once a month	29	61.7	3.39	1.00	11.54	0.050
Contact with the representatives less than once a month	18	38.3	1.00			
Contact with non-government organization representatives working in physical activity promotion						
More frequent (at least once a month)	22	46.8	0.57	0.17	1.91	0.367
Less frequent (less than once a month)	25	53.2	1.00			
Contact with international organizations representatives working in physical activity promotion						
More frequent (at least once a month)	23	48.9	3.35	0.77	14.58	0.107
Less frequent (less than once a month)	24	51.1	1.00			
Contact with GoPA! Country Contacts						
More frequent (at least once a month)	21	44.7	2.47	0.64	9.55	0.190
Less frequent (less than once a month)	26	55.3	1.00			
Country Cards provide information that is already known						
Agree and partially agree	15	31.9	0.33	0.09	1.17	0.086
Disagree	32	68.1	1.00			
I do not know what I am supposed to do with the Country Card						
Agree and partially agree	15	31.9	0.62	0.20	1.92	0.406
Disagree	32	68.1	1.00			
I do not know any strategy or how can I identify/reach partners/decision makers/stakeholders						
Agree and partially agree	17	37.0	0.74	0.23	2.35	0.613
Disagree	29	63.0	1.00			
The Country Card was useful and helped me making the case for physical activity promotion in my country						
Yes	45	95.7	32.49	5.22	202.21	< 0.001
No	2	4.3	1.00			
Country card completion						
Yes (all 5 indicators presented in the card)	32	37.2	1.49	0.46	4.77	0.178
No	15	26.3	1.00			
Country card indicators						
National physical activity policy						
Standalone policy for physical activity	25	53.2	2.72	0.82	9.04	
No standalone policy for physical activity	22	46.8	1.00			0.103
Physical activity surveillance						
Surveillance (at least one national survey including physical activity)	44	93.6	1.48	0.14	15.85	0.748
No surveillance (no national survey including physical activity)	3	6.4	1.00			
Research in physical activity						
Research in physical activity (at least one publication in 2013)	41	87.2	2.83	0.47	19.19	
No research (no publications in 2013)	6	12.8	1.00			0.287
Deaths due to physical inactivity						
Equal or more than the worldwide mean of deaths (9%)	27	57.5	2.16	0.41	11.26	0.738
Less than the worldwide mean of deaths (9%)	12	25.5	1.28	0.19	8.71	
No indicator	8	17.0	1.00			
Physical activity prevalence						
Has a national estimate	44	93.6	0.79	0.93	6.73	
Does not have a national estimate	3	6.4	1.00			0.829
World region^b^						
AFRO	3	6.4	0.15	0.06	3.85	0.030
EMRO	2	4.3	0.41	0.02	10.28	
EURO	19	40.4	1.00			
PAHO	17	36.2	4.77	0.84	27.03	
SEARO	2	4.3	11.98	1.64	87.37	
WPRO	4	8.5	1.48	0.30	7.44	
Income group						
High Income	29	61.7	1.00			
Upper Middle Income	12	25.5	0.73	0.01	39.08	0.940
Lower Middle Income	5	10.6	1.54	0.17	14.42	
Low Income	1	2.1	0.80	0.17	3.71	

^a^Adjusted by statistically significant variables in the unadjusted model (country contact status, contact with representatives (government, NGOs, International organizations, country contacts) opinions and barriers to country card use, and national policy indicator)

^b^EURO - European Regional Office of the World Health Organization; AFRO - African Regional Office of the World Health Organization; PAHO - Pan American Health Organization of the World Health Organization; EMRO - Eastern Mediterranean Regional Office of the World Health Organization; WPRO - Western Pacific Regional Office of the World Health Organization; SEARO - South-East Asia Regional Office of the World Health Organization

### Perceived barriers and opinions about the country cards

Perceived barriers to further use of the Country Cards are listed in Table 4. The most frequently reported barrier to Country Card use was that respondents did not know how to identify partners, decision makers or stakeholders (16.4%), followed by the lack of knowledge of what to do with the Country Card (15.1%).

**Table 4:** Country Cards barriers for use, opinions and suggested periodicity

	Total		GoPA! Country Contacts		ISPAH respondents (not GoPA! Country Contacts)	
	n	%	n^a^	%	n	%
Barriers to the use and dissemination of the Country Cards						
Country Cards provide information that is already known						
Agree and partially agree	15	15.5	7	19.4	8	13.1
Disagree	82	84.5	29	80.6	53	86.9
I do not know what I am supposed to do with the Country Card						
Agree and partially agree	21	22.6	1	3. 2	20	32. 3
Disagree	72	77.4	30	96.8	42	67.7
I do not know any strategy or how can I identify/reach partners/decision makers/stakeholders						
Agree and partially agree	23	24.7	6	17.6	17	28.8
Disagree	70	75.3	28	82.4	42	71.2
Opinions about the Country Card						
The Country Card provided new information and aroused interest						
Always	7	13.2	7	13.2	–	–
Frequently	41	77.4	41	77.4	–	–
Never	5	9.4	5	9.4	–	–
The Country Card was useful and helped me making the case for physical activity promotion in my country						
Always	19	14.7	12	22.6	7	9. 2
Frequently	77	59.7	32	60.4	45	59.2
Never	33	25.6	9	17.0	24	31.6

^a^n does not add to total value of 143 due to missing data

When analyzing barriers by region, respondents from EURO (57.0%) most frequently agreed that the information presented in the cards was already known followed by WPRO (38.5%), PAHO (33.0%), EMRO (25.0%) SEARO (20.0%) and, AFRO (18.2%). More than 50% of respondents from SEARO (80.0%) and WPRO (73.1%) agreed on a lack of knowledge of what to do with the card, followed by PAHO (42.9%), EURO (41.2%), AFRO (36.4%) and EMRO (25.0%). Also, respondents in SEARO (80.0%) predominantly agreed with not having strategies or knowledge to reach partners/decision makers or stakeholders, followed by EMRO (66.7%), WPRO (61.5%), AFRO (50.0%), EURO (48.1%) and PAHO (40.5%).

The open-ended responses provided insights into some of the barriers to Country Card use and varying opinions by region. For example, participants from the EURO region noted: *“The value of the Country Card (…) is limited because we have a very good information system in place, thus makes the added value of the card limited. However, it is of use in comparing my country with other countries”.* Respondents from high-income countries (mainly the EURO region) with established physical activity programs policies and with strong physical activity research and surveillance perceived that much of the information was already known and thus the Country Cards were viewed as less useful. A government representative questioned the data collection methods. *“The number of researchers in a country, publishing in the field of physical activity cannot be determined if publications in the local language are not considered”.* It was also noted that physical activity data provided on the Country Cards was not as relevant to the country*. “In my opinion, the physical activity data does not reflect the situation of our country. The official data on national physical activity is provided by our institution since 1984”. In order to reach high-level officials within each country it was suggested by one respondent that “the Country Cards should be disseminated in collaboration with the WHO”.*

However, opportunities for their use in advocacy for physical activity promotion were noted. *“We prepared a document on how to run a physical activity surveillance system (…). The Country Card was a good argument that surveillance is needed”. “I have primarily used it as an example of a good advocacy tool aimed at politicians and lay people/media”. “The main problem with answering these questions is that there are hardly any officials or professionals (except for those in the WHO country office) who are deemed in charge of physical activity related issues or NCDs in general”.*

In contrast, one respondent from AFRO reported that people were surprised to see the scarcity of national data on physical activity and the lack of research teams within the country. Another African respondent expressed disappointment that their country does not have data on the Country Card and this was thought to reflect the need for more research in the country around physical activity promotion *“My country still lacks Country Card details and this is really disappointing and I think a lot of research and publication needs to be done regarding Physical Activity promotion”. “There was a very good reception and interest, hard copies were distributed at the National Health Conference (2015) and discussed with the Ministry of Sports and Culture Physical Activity Unit (…) as well as verbally in meetings at the Faculty of Health Science”. “In general, the data regarding physical activity and health (…) create interest to those who see the data”.*

In two PAHO countries, GoPA! was identified as a critical factor for maintaining national physical activity surveillance efforts. In a country that was considering removing the physical activity module: *“The GoPA! Country Card was very useful in keeping the Global Physical Activity Questionnaire (GPAQ) in the National Health survey 2016–17”.*

Reactions from the WPRO Region highlighted the needs for both more accurate information and advocacy. *“Interest was shown by colleagues in academia, recognizing the requirement that surveillance systems use a standardized, validated assessment tool repeatedly and according to consistent protocols”. “The Country Cards have been used to advocate for physical activity”. “The Country Cards have been part of our advocacy of physical activity to the State Sports Administration (Ministry level)”.*

Respondents reported that a ranking of countries on physical activity prevalence would be a useful addition to the cards. One respondent pointed out the challenges in comparing nations due to the varied surveillance systems, but that the Country Cards help make the case for utilizing standardized measurers. Another suggestion was that the Country Cards should contain more detail on the initiatives to promote physical activity within each country.

Participants ranked (with 1 being not important at all and 5 being the most important) the physical activity policy indicator (weighted average 3.78) as most important for describing the physical activity status at the national level, followed by deaths due to physical inactivity (weighted average 3.73), national surveillance (weighted average 3.64), physical activity prevalence (weighted average 3.58) and research (weighted average 3.57).

## Discussion

To our knowledge, this is the first study evaluating the use of a standardized surveillance and advocacy tool such as the GoPA! Country Cards for global physical activity promotion. Key findings indicated that: 1. Being a country representative working in academia and reporting collaboration with a government representative working in NCDs were factors associated with more diverse and frequent use of the Country Cards; 2. The perception of the relevance and usefulness of the Country Cards was greater in low- and middle-income countries than in high-income countries; 3. Country Cards were used in at least half of their possible applications, and specific uses of Country Cards varied by World Bank income group, world region and country-level capacity for physical activity promotion; and, 4. We identified gaps in knowledge and use of Country Cards, providing important information for guiding actions to optimize physical activity promotion, surveillance and research efforts at the national, regional, and global levels.

The fact that GoPA! country representatives were the main users of the Country Cards highlights the importance of engaging local actors from the early stages of the development process of standardized global surveillance initiatives such as GoPA!. Early engagement with the end-users of this advocacy tool appears to have led to a greater familiarity, understanding and use of the Country Cards for physical activity promotion. Among users reporting frequent and/or diverse use of GoPA! Country Cards, a substantial proportion had an academic background. In fact, one of the most common ways in which Country Cards were reported to be used was for the development of academic products (peer-reviewed research articles, research proposals, presentations at scientific conferences, and doctoral dissertations). This is not surprising, since GoPA! Country Cards are an evidence-based promotion and advocacy tool likely to appeal especially to academics.

The most positive perceptions of the relevance and usefulness of the Country Cards was reported by users from low- and middle income countries. This is an important finding, as the majority of the world’s population lives in these countries with a high NCD burden. These settings also tend to have low capacity for physical activity research and surveillance relative to high-income settings. Our results suggest that it is precisely in these settings with high need and low capacity where GoPA!‘s Country Cards have the greatest potential for positively influencing physical activity promotion and policy [13]. These countries may benefit from new sets of Country Cards to assist in the evaluation of surveillance, research and promotion efforts in coming years. On the other hand, for high-income countries with a higher baseline level of research and surveillance capacity in this field, GoPA! Country Cards may represent a useful tool to complement or optimize existing efforts.

The fact that Country Cards were used in at least half of their possible applications varying by income group, region and country-level capacity for physical activity promotion may be due to the short time between the launch of the Country Cards and survey data collection, which may not have provided sufficient time for full uptake and use of the cards. Also, country contacts came from variable sectors, thus differences in knowledge and use of the country cards may not reflect differences in the countries, but differences in the respondent’s situation and perspective.

Up to this point, the GoPA! Country Cards appear to be providing the evidence and messaging for the first (why) and the second (what) steps in the three step model of advocacy for physical activity promotion [14]. Targeted efforts such as the GoPA! pyramid and stages of country-level capacity (Fig. 1) and, the recommended activities for physical activity promotion based on the Country Card indicators (Table 5), are now available and could help optimize use of the cards for the third step of advocacy “HO*W*/WHO” allowing policy makers and government representatives to get involved, plan a strategy and improve national capacity for physical activity promotion.

**Table 5:** Steps to achieve high country-level capacity for physical activity promotion according to country stage based on the GoPA! pyramid for country-level capacity (Fig. 1)

	Stage 1	Stage 2	Stage 3
Activities recommended according to the stages of country-level capacity for physical activity promotion based on GoPA! Country Card indicators (Fig. 1b) (Activities are listed in hierarchical order)			
Country Contacts	1. Estimate the magnitude of the problem. 2. Identify and support research groups. 3. Report the magnitude of the problem, and identify groups and regions at higher risk. 4. Use surveillance and research data to make the case for a stand-alone national physical activity policy document. 5. Use the GoPA! Country Card to encourage strategic partners to start building the pyramid for country-level capacity. 6. Use the GoPA! “1st Physical Activity Almanac” to identify a) other stage 1 countries, and b) stage 2 and 3 countries – connect with them to problem-solve and develop strategies for pyramid improvement. 7. Strengthen regional capacity by reaching out to geographic neighbors. 8. Set a realistic timeline, with specific objectives. 9. Contact policy makers and researchers to disseminate the Country Card and encourage specific actions.	1. Maintain the indicators that were identified as high. 2. Improve the indicators that were set as medium and low and address specific gaps. 3. Address dissemination gaps. 4. Approach policy makers with the Country Card to make the case for HEPA promotion and to strengthen local capacity. 5. Support countries in Stage 1. 6. Contact policy makers and researchers at the country level to disseminate the Country Card and encourage specific actions.	1. Maintain and scale up the pyramid. 2. Identify and address dissemination gaps. 3. Focus on an integrated and multidisciplinary collaboration to translate research into policy and to scale up interventions that can lead to equity, social justice. 4. Approach policy makers with the Country Card and continue making the case for physical activity promotion to sustain and expand local capacity. 5. Set more ambitious goals and concrete timelines to achieve them to strengthen the pyramid. 6. Support countries in stages 1 and 2 by sharing experiences in developing and maintaining the pyramid. 7. Contact policy makers and researchers at the country level to disseminate the Country Card and encourage specific actions.
Government and policy	1. Support the creation of a national physical activity surveillance system through legislative and budgetary actions. 2. Stimulate national physical activity research: provide funds/incentives for physical activity training programs and capacity building. 3. Clearly outline political commitment to and resources for physical activity, establish multi-sectoral approaches. 4. Review financial and other resources available to implement and monitor appropriate PA policies. 5. Engage in fund raising for physical activity policy implementation.	1. Cooperate with ministries across multiple sectors. 2. Initiate a collective meeting with governmental representatives from the transport, housing, health, infrastructure, urban design, planning, environment, sports and recreation and education sectors and present the Country Card as an evidence-based physical activity resource. 3. Ensure availability of financial and other resources to implement and monitor appropriate physical activity policies.	1. Discuss and arrange the implementation of actions geared at sustaining, strengthening, and scaling-up the three pillars, at national, regional and local level. 2. Maintain and expand financial commitment to implement and monitor physical activity policies. 3. Strive for equity, by reducing social and health inequalities of access to opportunities for physical activity.
Researchers	1. Critically evaluate the data sources for your Country Card and update as needed. 2. Identify any local capacity to start high-quality physical activity research. 3. Raise awareness and present the Country Cards to colleagues and students, stressing the gaps identified and the potential to drive a new field of work nationally. 4. Bring attention to GoPA! through dissemination using existing networks.	1. Build physical activity capacity and support further training in research, practice, policy, evaluation and surveillance. 2. Identify any existing networks, or start one (if necessary). Promote collaboration across research groups with physical activity capacity in the country. 3. Bring attention to GoPA! through dissemination using existing networks.	1. Produce research linking GoPA! to the needs, actions and goals of primary health care, transport, housing, health, infrastructure, urban design, and education sectors. 2. Use key supplemental resources to stress the health benefits of physical activity (Lancet Physical Activity series, Bangkok Declaration, Global Action Plan for Physical Activity), and to guide new research.

Finally, gaps in knowledge about the content, potential uses, and ways to distribute and promote and the Country Cards were identified as critical challenges which must be addressed to guide further actions with the Country Cards. The data sources for the indicators included in the Country Cards (described in the Country Card appendix and GoPA! website) were not always well-known to the respondents. A possible explanation for this could be the limited dissemination of the Country Cards outside of the GoPA! network. Some respondents that were familiar with the WHO Country Fact Sheets and with the WHO Global Health Observatory [15] were confused by the discrepancies between the prevalence of physical activity reported by the WHO Global Health Observatory and that of GoPA!. While WHO presents the prevalence of physical inactivity using the last edition of the WHO STEPS surveillance survey, GoPA! Country Cards use the most recent prevalence of physical activity available from either the national surveillance system of each country, or by recalculating the data from the WHO Global Health Observatory to obtain the prevalence of meeting international physical activity recommendations [15]. GoPA!‘s use of the most recent and best available country-level data means that there will occasionally be differences from the WHO data bases [1].

Although many respondents reported that the Country Cards provided a succinct approach to presenting the global perspective of physical activity to various audiences, lack of skills to effectively use the Country Cards as an advocacy tool was identified as a critical barrier for their widespread use. In response, we have identified a sequence steps for countries to achieve high capacity for physical activity promotion, depending on the current stage of each country (see the GoPA! pyramid for country-level capacity in Fig. 1, and steps for increasing capacity in Table 5). Table 5 includes activities for optimizing the use of the Country Cards by countries. We believe that this resource can accelerate the process of increasing country-level capacity for physical activity promotion. Further steps to reduce knowledge gaps should include targeted training efforts to maximize Country Card use, particularly in low- and middle-income countries. These efforts should include strategic dissemination methods and the development and use of additional supporting materials, some of which are available on the GoPA! website [7].

### Limitations

Results should be interpreted with caution given the following limitations. The cross sectional nature of the study limits the ability to establish causality and, it may be possible that associations are due to chance given that the analyses were conducted using a relatively small sample. The generalizability of the results may be diminished by the low response rate and variability by geographic region. The low survey response rate may be due to the short time that the survey was open for response (two months). Previous studies have shown similar or lower response rates for internet-based surveys, especially as compared to traditional survey methods. This is thought to be due to differences in the use of incentives, mode of contact, varying internet access, and the number of contact attempts [16].

## Conclusion

Our study demonstrated that the relevance and usefulness of GoPA! Country Cards was associated with being part of the GoPA! network, knowing about the GoPA! Country Cards, living in low- and middle-income countries, and on the stage of country capacity for physical activity promotion. GoPA’s Country Cards may prove to be a critical strategy for tipping the scale in favor of PA promotion, research and surveillance strategies in these countries (LMICs) where historically the recognition of inactivity as a public health problem, as well as the available local capacity to study it, measure it, and promote it, have been quite limited. For the Country Cards to have a broader impact on physical activity promotion and NCD prevention, GoPA! will need to widen its reach beyond the academic sector and target countries with limited capacity for physical activity promotion. Further refinement of the cards and training in their use can be an important tool for advancing country capacity for contextually-relevant strategies, actions and timelines for PA promotion. As a council of the International Society of Physical Activity and Health (ISPAH), GoPA! supports existing global efforts such as the Toronto Charter for Physical Activity and The Bangkok Declaration for Physical Activity [17, 18] and is contributing to the WHO Global Action Plan for Physical Activity -GAPPA [4] to facilitate coherent global efforts for increasing physical activity promotion and advocacy.

## Additional file

Additional file 1: List of 139 GoPA! members by August 2016 (in bold the new GoPA! members up to September 2017 for a total of 144 GoPA! members). (DOCX 16 kb)
