# Supplementary material for: Worldwide use of the first set of physical activity Country Cards: The Global Observatory for Physical Activity - GoPA!
Source: Int J Behav Nutr Phys Act. 2018 Mar 27;15:29. doi: 10.1186/s12966-018-0663-7 (PMC5869782; doi:10.1186/s12966-018-0663-7)
Supplement: Supplementary file 1 — List of 139 GoPA! members by August 2016 (in bold the new GoPA! members up to September 2017 for a total of 144 GoPA! members). (DOCX 16 kb) [file 12966_2018_663_MOESM1_ESM.docx]

**Additional file 1: List of 139 GoPA! members by August 2016 (in bold the new GoPA! members up to September 2017 for a total of 144 GoPA! members).**

American Samoa, Angola, Antigua and Barbuda, Argentina, Aruba, Australia, Austria, Bahrain, Bangladesh, Barbados, Belgium, Bermuda, Bhutan, Bolivia, Botswana, Brazil, Brunei Darussalam, Bulgaria, Cameroon, Canada, Cayman Islands, Chile, China, Colombia, Costa Rica, Croatia, Cuba, Czech Republic, Denmark, Dominica, Dominican Republic, Ecuador, Egypt, England, Estonia, Faeroe Islands, Fiji, Finland, France, French Polynesia, **Gambia,** Germany, **Georgia,** Ghana, Greece, Greenland, Grenada, Guam, Guatemala, Guyana, Haiti, Hong Kong SAR China, Hungary, Iceland, India, Indonesia, Iraq, Ireland, Israel, Italy, Japan, Jordan, Kazakhstan, Kenya, Kiribati, Korea Republic, Lao PDR, Lebanon, Lithuania, Luxembourg, Macao SAR China, Macedonia, Malaysia, Maldives, **Mali,** Malta, Marshall Islands, Mexico, Micronesia, Moldova, Mongolia, Mozambique, Myanmar, Nepal, Netherlands, New Caledonia, New Zealand, Nicaragua, Nigeria, Northern Ireland, Northern Mariana Islands, Norway, Oman, Palau, Palestine/West Bank and Gaza, Papua New Guinea, Paraguay, Peru, Poland, Portugal, Puerto Rico, Qatar, Romania, Russian Federation, Rwanda, Samoa, Saudi Arabia, Scotland, Senegal, Seychelles, Singapore, Slovak Republic, Slovenia, Solomon Islands, **Somalia,** South Africa, Spain, Sri Lanka, St. Kitts and Nevis, St. Lucia, St. Martin (French part), St. Vincent and the Grenadines, **Suriname,** Swaziland, Sweden, Switzerland, Syrian Arab Republic, Tanzania, Thailand, Tonga, Trinidad and Tobago, Tunisia, Turkey, Tuvalu, Uganda, Ukraine, United Arab Emirates, United States, Uruguay, Vanuatu, Venezuela, Vietnam, Virgin Islands, Wales.
